# Psychological Profiles of Chinese Patients With Hemodialysis During the Panic of Coronavirus Disease 2019

**DOI:** 10.3389/fpsyt.2021.616016

**Published:** 2021-03-03

**Authors:** Zhen-Hua Yang, Xiao-Ting Pan, Yu Chen, Lu Wang, Qiu-Xin Chen, Yan Zhu, Yu-Jia Zhu, Yong-Xi Chen, Xiao-Nong Chen

**Affiliations:** Department of Nephrology, Ruijin Hospital Affiliated to Shanghai Jiaotong University, School of Medicine, Shanghai, China

**Keywords:** hemodialysis, quality of life, mental health, psychological profiles, stress, COVID-19

## Abstract

**Background:** Hemodialysis patients not only suffer from somatic disorders but are also at high risks of psychiatric problems. Early this year, the outbreak of coronavirus disease 2019 (COVID-19) has caused great panic and anxiety worldwide. The impact of this acute public health event on the psychological status of hemodialysis patients and its relationship with their quality of life have not been fully investigated.

**Methods:** This study comprised two parts. The initial study enrolled maintenance hemodialysis patients treated in Ruijin Hospital for more than 3 months from March to May 2020 during the ongoing COVID-19 pandemic. Patients completed three questionnaires including the Impact of Events Scale–Revised (IES-R), General Health Questionnaire-28 (GHQ-28), and Kidney Disease Quality of Life (KDQOL) Short Form (SF). Follow-up study was performed from December 2020 to January 2021, when the pandemic of COVID-19 has been effectively contained in China. Only patients enrolled in the initial study were approached to participate in the follow-up study.

**Results:** There were 273 maintenance dialysis patients enrolled in the initial study and 247 finished the follow-up study. For the initial study, the estimated prevalence of nonspecific psychiatric morbidity was 45.8% (125/273) by GHQ-28. By IES-R, 53/273 (19.4%) patients presented with total scores above 24 that reflected clinical concerns. We found a significant difference regarding KDQOL scores between patients with different stress response (IES-R) groups (*p* = 0.026). Our follow-up study showed that KDQOL and SF-36 scores were significantly improved in comparison with those in the initial study (*p* = 0.006 and *p* = 0.031, respectively). Though total scores of GHQ-28 and IES-R did not change significantly, some subscales improved with statistical significance. Furthermore, gender, education background, and duration of hemodialysis were three factors that may affect patients' mental health, quality of life, or health status while dialysis duration was the only variable that correlated with those parameters. However, these correlations were combined effects of the COVID-19 pandemic and the dialysis itself.

**Conclusions:** We found a correlation between changes in the mental health status of dialysis patients and changes in their quality of life. These responses were also mediated by patients' psychosocial parameters. Our results urge the necessity of psychotherapeutic interventions for some patients during this event.

## Introduction

Chronic kidney disease (CKD) is now a global health problem that affects one out of 10 adults worldwide (1, 2). In China, the overall prevalence of CKD was about 10.8% in 2012 (3) and the figure is still increasing. Regardless of the pathogenesis of the disease, the progression of CKD would ultimately lead to end-stage renal disease (ESRD)—a devastating disease that requires dialysis or transplantation in some patients. The impact of ESRD is huge not only in terms of its repercussions on patients but also its burden on the health resources.

In addition to somatic disorders caused by the disease and its complications, ESRD patients also experience high prevalence of psychiatric problems (4, 5). Anxiety or depression occurs in ~10–45% of patients with hemodialysis (6–8). These mental disorders would cause not only non-compliance to treatment but also severe consequences. Consequently, mental health problems in these patients are closely associated with their morbidity and mortality (9, 10). Moreover, psychological variables and aspects of the social environment add much difficulty to the management of their psychological disorders because these factors are intersecting and complex. Given this background, investigating psychosocial factors affecting ESRD patients would provide us with knowledge to identify and manage psychiatric problems in this population.

Psychosocial factors are a vast number of intersecting variables that include individual demographic features, psychologic and behavioral characteristics, social or environmental factors, and patient-level variables. Any factors causing failure of these variables to return to normal would lead to abnormality of the allostatic system and result in psychological disorders in patients. In early 2020, the outbreak of the novel coronavirus disease 2019 (COVID-19) has caused great panic and anxiety worldwide. The pandemic nature of the disease makes vulnerable populations at high risk of infection and causes great stress among patients with hemodialysis. However, the impact of this acute public health event on the psychological status of those patients has not been fully investigated. In this study, we focus on the psychological profiles of patients with hemodialysis in this event to provide a better understanding of the influence of psychosocial factors on the mental health of this population.

## Materials and Methods

### Patients

The initial study was performed between March and May 2020 in the hemodialysis center of Ruijin Hospital affiliated to Shanghai Jiaotong University School of Medicine to study the psychological profiles of the patients during ongoing COVID-19 pandemic. All patients under hemodialysis therapy for at least 3 months were approached to participate in the initial study. The follow-up study was performed between December 2020 and January 2021 to compare the psychological profiles of the patients after COVID-19 pandemic. Only patients enrolled in the initial study were approached to participate in the follow-up study.

### Measure

Patients completed three validated questionnaires, including the revised version of Impact of Events Scale, General Health Questionnaire-28, and Kidney Disease Quality of Life Short Form.

#### Impact of Events Scale–Revised

The Impact of Events Scale–Revised (IES-R) is a 22-item self-report instrument assessing subjective distress resulting from everyday trauma or acute stress. We adapted IES-R to assess the presence and severity of psychological symptoms experienced by subjects at any time during the current acute public events. Likert rating scale from 0 to 4 was used for each item of IES-R, and the total score was 0 to 88. Total scores of IES-R that exceed 24 reflect clinical concern (11), scores above 33 reflect a probable diagnosis of post-traumatic stress disorder (PTSD) (12), and scores above 37 reflect suppression of immune system function (13). The IES-R has been translated into Chinese and validated in literature (14, 15).

#### General Health Questionnaire-28

The General Health Questionnaire-28 (GHQ-28) is a 28-item screening tool to detect non-specific psychiatric disorders among individuals in primary care settings (16, 17). GHQ-28 is designed to measure mental health disorders and could be grouped into four subscales: somatization, anxiety, social dysfunction, and depression. Each item is assessed using the 0-0-1-1 scoring method. The total score on the GHQ-28 ranges from 0 to 28 (18). We adopted a cutoff score of 12 out of 28 (those who answered positively to 12 questions would be considered a “case”) (18).

#### Kidney Disease Quality of Life Short Form

The Kidney Disease Quality of Life Short Form (KDQOL-SF), which has been used in ESRD patients widely, assesses the quality of life of patients with kidney diseases (19). KDQOL-SF comprises 43 disease-specific items (symptoms/problem list, effects of kidney disease, burden of kidney disease, work status, cognitive function, quality of social interaction, sexual function, sleep, social support, dialysis staff encouragement, and patient's satisfaction), 36 generic items (physical functioning, role—physical, pain, general health, emotional well-being, social function, and energy/fatigue), and background information. KDQOL-36 has been translated and validated in Chinese population (20).

### Statistical Analyses

Statistical analysis was performed using SPSS 13.0 (SPSS Inc.). Data with normal distribution were summarized as mean ± SD. Data without normal distribution were summarized as median. Comparisons were made using the Student *t*-test or one-way ANOVA for continuous variables and the χ^2^-test for categorical variables as required. Pearson correlations were derived, and tests of significance were set at 0.05. Multiple regression analysis was used to analyze association between different variables.

## Results

### Demographic Features

**Figure 1:**
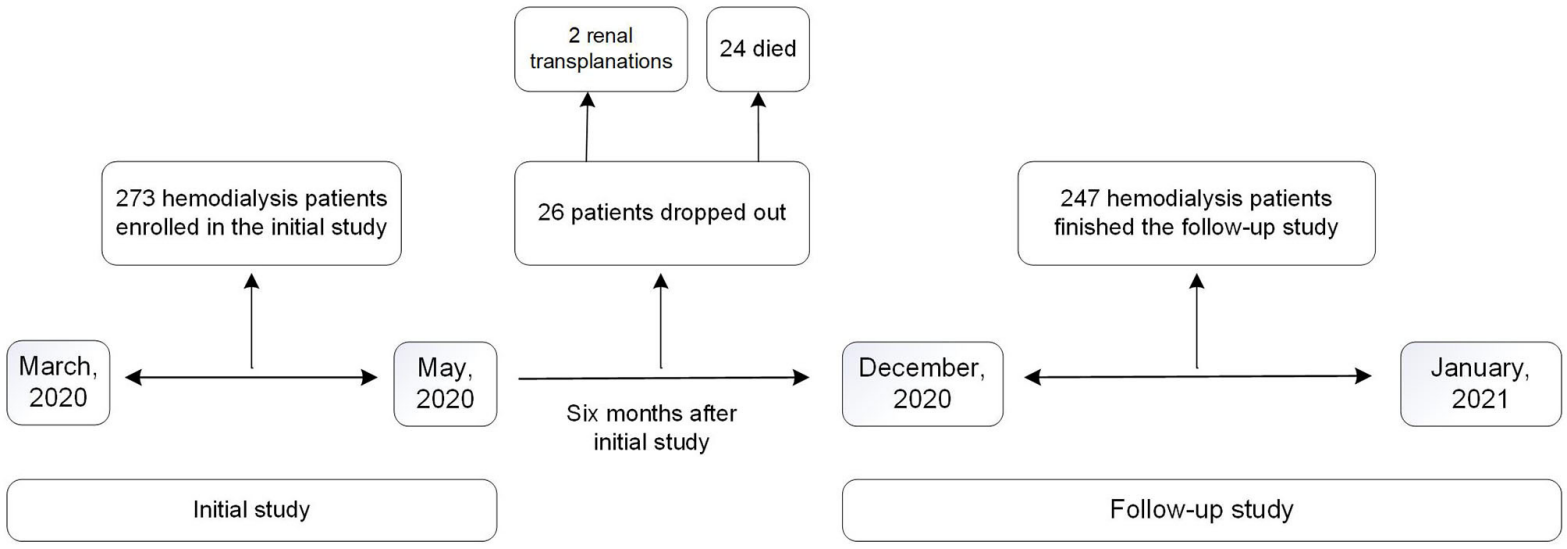
Flow diagram of study design.

The flow diagram of study is demonstrated in Figure 1. There were 273 maintenance dialysis patients enrolled in the initial study. Male patients composed 58.6% of all the patients, and primary glomerulonephritis was the most common cause (71.8%) of ESRD. Majority of the patients (70.3%, 192/273) received education of secondary or less. At the time of survey, only 16.1% (44/273) of the patients had full or part time job. The baseline characteristics are summarized in Table 1. During follow-up, two patients received renal transplantation and 24 died; the remaining 247 patients finished the follow-up study.

**Table 1:** Baseline characteristics of the patients.

**Characteristics**	**Hemodialysis patients (*n* = 273)**
Age (years, mean ± SD)	59.9 ± 14.4
Gender (female/male)	113/160
Duration of hemodialysis (months, mean ± SD)	78.7 ± 60.5
Marital status (*n*, %)	
Married	219 (78.5%)
Divorced or widowed	23 (8.2%)
Single	31 (11.1%)
Education background (*n*, %)	
Primary or less	19 (7.0%)
Secondary	173 (63.4%)
University or higher	81 (29.7%)
Etiology of end-stage kidney disease (*n*, %)	
Diabetic kidney disease	32 (11.7%)
Primary glomerulonephritis	196 (71.8%)
Renal vascular disease	20 (7.3%)
Others	25 (9.2%)

### Mental Health and Quality of Life

Table 2 summarizes the psychological profiles (GHQ-28, IES-R) and quality of life of the patients (KDQOL and SF-36) during the initial study and follow-up study.

**Table 2:** Psychological profiles, metal health, and quality of life of the patients.

**Variables**	**Initial study**	**Follow-up study**	***p***
	**(*n* = 273)**	**(*n* = 247)**	
Quality of Life (KDQOL) (mean, 95% CI)	60.2 (59.0–61.3)	63.4 (61.9–65.0)	0.006
Health status (SF-36) (mean, 95% CI)	59.6 (57.4–61.8)	62.8 (60.5–65.1)	0.031
GHQ-28 score (mean, 95% CI)	13.1 (12.0–14.2)	12.2 (11.2–13.1)	NS
Somatic symptoms	3.7 (3.5–4.0)	3.1 (2.8–3.4)	0.006
Anxiety and insomnia	2.8 (2.4–3.1)	1.9 (1.7–2.2)	0.005
Social dysfunction	4.5 (4.2–4.8)	4.7 (4.4–5.1)	NS
Depression	2.2 (1.8–2.5)	2.4 (2.0–2.7)	NS
IES-R score (mean, 95% CI)	13.4 (11.8–15.0)	13.1 (11.4–14.8)	NS
Intrusion score	5.0 (4.4–5.6)	4.8 (4.2–5.5)	0.049
Avoidance score	4.8 (4.2–5.4)	4.4 (3.7–5.1)	NS
Hyperarousal score	3.6 (3.2–4.1)	3.9 (3.2–4.1)	NS

*KDQOL, Kidney Disease Quality of Life; SF-36, Short Form-36; 95% CI, 95% confidence interval; GHQ-28, General Health Questionnaire-28; IES-R, Impact of Event Scale–Revised*.

*KDQOL-SF comprises 43 disease-specific items (KDQOL) and 36 generic items (SF-36)*.

*GHQ-28 consists of four subscales: somatic symptoms, anxiety and insomnia, social dysfunction, and depression. The score range of each subscale is 0–7 and the total GHQ-28 score range is 0–28. The higher score represents more severe mental health disorders*.

*IES-R consisted of three subscales: avoidance (range 0–28), intrusion (0–32), and hyperarousal (0–24). The total score of IES-R ranged from 0 to 88. Total scores of IES-R that exceed 24 reflect clinical concern (11), scores above 33 reflect a probable diagnosis of PTSD (12), and scores above 37 reflect suppression of immune system function (13)*.

The initial study, which was performed in the ongoing COVID-19 pandemic, showed the total score of GHQ-28 was 13.1 in our patients. A higher score signifies a greater number of symptoms, and details of GHQ subscales are summarized in Table 2. By adopting a cutoff score of 12 out of 28 (18), we found an estimated prevalence of non-specific psychiatric morbidity of 45.8% (125/273). Furthermore, the mean scores for social dysfunction and somatic symptoms were higher compared with the mean scores for anxiety and insomnia and for depression.

Total score and scores of subscales of IES-R are also shown in Table 2. In our study, 53/273 (19.4%) patients presented with total scores above 24, which reflected clinical concerns. Among those patients, 5/273 (1.8%) patients had a probable diagnosis of post-traumatic stress disorder (PTSD) with score >33, and 29/279 (10.6%) patients had scores above 37, which reflected the suppression of immune system functioning.

The follow-up study showed that KDQOL and SF-36 scores significantly improved compared with those in the initial study (*p* = 0.006 and *p* = 0.031, respectively), which suggested the improved quality of life after COVID-19 pandemic in our patients. We also compared the total GHQ-28 score and IES-R score. Though total scores of these two scales showed no significant difference, improved somatization symptoms, anxiety and insomnia, and intrusion subscales were found in the follow-up study (*p* = 0.006, *p* = 0.005, and *p* = 0.049, respectively).

### Comparison of Quality of Life Between Different Psychiatric Diagnostic Groups During Ongoing COVID-19 Pandemic

We divided the patients into different psychopathology groups according to their IES-R scores or GHQ-28 scores to investigate the interplay between psychiatric diagnosis and quality of life during ongoing COVID-19 pandemic.

By adopting a cutoff of 12 out of 28 by GHQ-28, we did not find any significant difference regarding KDQOL or SF-36 between patients with non-specific psychiatric disorders (GHQ-28 score ≥12) and those without (*p* > 0.05, data not shown). If we divided patients based on IES-R scores, we found a significant difference regarding KDQOL between different groups (*p* = 0.026) (Table 3). Furthermore, four subscales of KDQOL (work status, cognitive function, quality of social interaction, and sleep) were found significantly different (*p* = 0.027, *p* = 0.022, *p* = 0.010, and *p* = 0.039, respectively). However, we did not find a significant difference regarding SF-36 and its subscales between different groups.

**Table 3:**
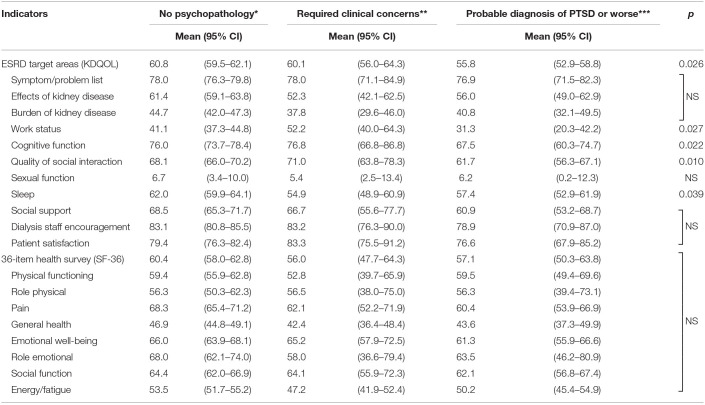
Effects of acute stress on quality of life of the patients during ongoing COVID-19 pandemic.

*ESRD, end-stage renal disease; PTSD, post-traumatic stress disorder; KDQOL, Kidney Disease Quality of Life; SF-36, Short Form-36; 95% CI, 95% Confidence interval*.

*Patients with IES-R ≤ 24 (11).

**Patients with IES-R >24 but IES-R <33 (11, 12).

***Patients with IES-R ≥33 (12).

### Effects of Demographic and Exposure Variables on Mental Health and Quality of Life During Ongoing COVID-19 Pandemic

Table 4 summarizes the effects of demographic factors on patients' mental health status and quality of life. We presented the results of GHQ-28, IES-R, KDQOL, and SF-36 total score as measures in relation to various demographic experiences. Our results showed that gender, education background, and duration of hemodialysis were three important factors that may affect patients' mental health, quality of life, or health status.

**Table 4:**
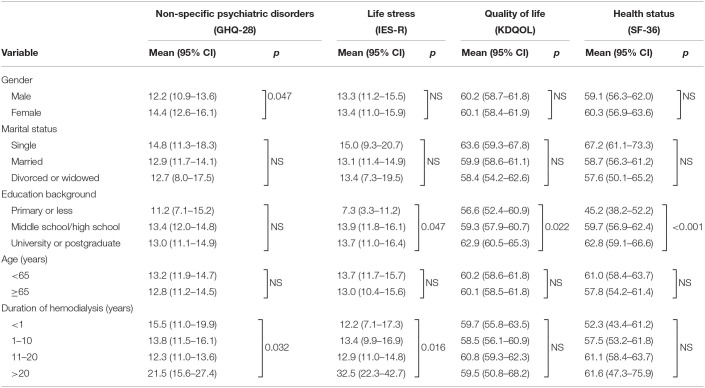
Effects of demographic and exposure variables on mental status and quality of life of the patients during ongoing COVID-19 pandemic.

*GHQ-28, General Health Questionnaire-28; IES-R, Impact of Event Scale–Revised; KDQOL, Kidney Disease Quality of Life; SF-36, Short Form-36; 95% CI, 95% confidence interval*.

### Utility of Mental Health and Quality of Life

In our study, GHQ-28 was correlated to IES-R, which suggested patients' mental health status was correlated to their stress response (Table 5). Similarly, KDQOL was also correlated to SF-36, suggesting quality of life and health status were both correlated. Furthermore, IES-R was negatively correlated with KDQOL with statistical significance, which suggested patients' quality of life was negatively affected by their distress from acute events.

**Table 5:** Correlation coefficients for the GHQ-28, IES-R, KDQOL, and SF-36 during ongoing COVID-19 pandemic.

	**GHQ-28**	**IES-R**	**KDQOL**	**SF-36**
GHQ-28	–	0.584^*^	−0.056	0.013
IES-R		–	−0.119^**^	−0.078
KDQOL			–	0.596^*^
SF-36				–

* p < 0.001;

** *p < 0.05*.

*GHQ-28, General Health Questionnaire-28; IES-R, Impact of Event Scale–Revised; KDQOL, Kidney Disease Quality of Life; SF-36, Short Form-36.s*.

We also analyzed the results of total scores of GHQ-28, IES-R, and KDQOL-SF in relation to various demographic variables. Our results showed KDQOL and SF-36 were both intercorrelated. Furthermore, dialysis duration was the only variable that correlated patients' mental health status (GHQ-28), response to stress (IES-R), and health status (SF-36). The correlation of other variables is summarized in Table 6.

**Table 6:** Multiple regression of dependent variables and related factors during ongoing COVID-19 pandemic.

**Variable**	***r*^**2**^**	***t***	**Final β**	***p***
Dependent variables: SF-36 score (constant)		−0.850		0.396
Dialysis duration of hemodialysis	3.202	2.376	0.116	0.018
KDQOL score	1.036	11.005	0.556	<0.001
Dependent variable: KDOQL score (constant)		8.960		<0.001
SF 36 score	0.304	11.005	0.565	<0.001
Dependent variable: GHQ-28 (constant)		2.095		0.037
Gender	1.879	2.011	0.102	0.045
Dialysis duration of hemodialysis	−1.473	−2.034	−0.107	0.043
Dependent variable: IES-R (constant)		1.265		0.207
SF-36 pain	−0.117	−2.455	−0.186	0.015
Dialysis duration of hemodialysis	2.297	2.221	0.113	0.027

*GHQ-28, General Health Questionnaire-28; IES-R, Impact of Event Scale–Revised; KDQOL, Kidney Disease Quality of Life; SF-36, Short Form-36*.

## Discussion

Psychological disorders among dialysis patients are not simply a consequence of short-term adjustment reaction to regimens but a long-term concomitant of coping with chronic dialysis and ESRD complications. In a recent cohort study, 22% of patients receiving maintenance hemodialysis had anxiety symptoms and 42% had depressive symptoms. In our study, the estimated prevalence of non-specific psychiatric morbidity was 45.8% by GHQ-28. Furthermore, the psychological disorders are closely associated with all-cause mortality and prolonged hospitalizations (21). Both our results and data from the literatures suggest the mental health disorders among dialysis patients are prevalent, which require timely diagnosis and adequate intervention so as to reduce mortality and improve prognosis.

Many factors contribute to poor mental health status among dialysis patients. Psychosocial parameter is one of such key factors. According to definition, it refers to a group of psychological variables and aspects of social environment that are central to individual's perception of quality of life (9). By adding burden of existing mental health status, psychosocial parameter could worsen patients' psychological status. Meanwhile, patients' perception accompanying the stressor could influence their functional status and eventually affect their prognosis (9). In our study, the average score of KDQOL and SF-36 was higher than those reported by Spain and US (22, 23). Such difference might reflect the influence of current acute public events on patients' quality of life. By further comparison of initial study, our follow-up study demonstrated that KDQOL and SF-36 scores as well as some subscales of GHQ-28 and IES-R were significantly improved after pandemic of COVID-19. Since disease itself and mitigation strategies during COVID-19 pandemic like home isolation, intense health monitoring, and many others would greatly affect dialysis patients' daily lives and access to dialysis therapy, our results thus suggest patients' quality of life and their mental health is greatly influenced by social environmental factors. It was also shown in the current study that gender and education background were two parameters associated with patients' mental health status as well as kidney disease quality of life. Education background determines patients' knowledge and perception to social environmental variables and compliance to renal replacement therapy, while gender is closely associated with other psychosocial factors like employment, income, education, and more; these intersected variables would consequently affect patients' mental health status and their physical well-being. Studies pointed out that psychosocial factors could affect patients' outcome by several mechanisms, which included access to health care, compliance with the dialysis therapy, and their health status (24). Our results thus suggest patient-level psychosocial parameters should receive special attention especially during stressing events as they could affect patients' mental health status as well as their kidney disease quality of life.

Though the prevalence of mental health disorders among dialysis patients is high, they are difficult to identify especially in patients with the backdrop of chronic dialysis. Overlap between uremic symptoms resulted from inadequate dialysis, and depressive symptoms add much difficulty to distinguish and manage dialysis patients with psychological disorders. One possible way to differentiate between psychiatric illness and medical illness is to delineate differences in thinking styles (25). By using professional tools like Diagnostic and Statistical Manual of Mental Disorders (DSM), patient's psychological disorders could be differentiated from mental health problems stemming from medical illness (25, 26). However, these tools are professional and sophisticated, which prevent them from being widely used in clinical practice. An alternative method to evaluate patients' psychological status is to use self-reporting screening tool that does not require professional knowledge to interpret. In our study, we adopted GHQ-28 to detect non-specific psychiatric morbidities among our patients. Results showed scores of social dysfunction and somatic symptoms were higher than anxiety or depression in our patients. Though the subscales of GHQ-28 are not designed to make a psychiatric diagnosis, these scores provide information for somatic, anxiety, social dysfunction and severe depression symptoms. Our results thus imply more attention should be paid to patients' social deficits as they may require more clinical concerns.

We also investigated patients' psychological response to acute stress during the current pandemic event. Stress indicates the change in the physical condition, environment, or psychosocial setting of an organism. It refers to the ability to achieve stability through changes. Failure of levels of stress mediator to return to baseline after challenge would cause abnormality of stress response. Since stressor and functional status of the subjects are two fundamental determinants of stress outcome (9), any changes in patient's status in personal or social contexts could result in depression, anxiety, or development of other mental health problems. By using IES-R, we investigated the psychological symptoms relating to various types of event exposure. Our results indicated that exposure to current acute stress and related events like home isolation, being quarantined, contact tracing, and many others contributed to the psychological symptoms of the patients with dialysis. Similar results were also reported by Wu and colleagues (14) who investigated psychological status of healthcare workers exposed to SARS-related events and found post-traumatic stress (PTS) symptom levels were closely associated with the outbreak of the disease and people's perception levels of the events were related to symptom levels.

We found that IES-R score was negatively correlated to KDQOL score in the current study. IES-R score was adopted for subjective distress from acute stress, and the higher score represented for the more severe psychological symptoms. The way patients respond to the stress would affect their perception and consequently influence their medical outcomes. Therefore, patients with higher IES-R score would have lower level of kidney quality of life. In a recent multicenter study, García-Martínez and colleagues (23) found that patients' resilience to stress was associated with their quality of life. Their results are consistent with our findings, which suggest patients' response to stress would have an impact on different aspects of their quality of life. In light of the important role of patients' response to acute stress, improving their resilience and coping capability with acute stress would help to increase quality of life and decrease the frequency of hospitalization in patients with hemodialysis (27–29).

There are several limitations that must be acknowledged in the current study. First, we did not provide historical profiles of the patients as controls because many patients began their dialysis therapy long before current evaluating tools were introduced in China. We therefore performed the follow-up study when COVID-19 pandemic was effectively contained and made the comparison. Second, our hospital is located in the downtown of the city and most of our patients are from urban areas. Considering social economic status, education background, and some other variables are different between urban and rural areas, data in the current study might not fully represent those from rural areas. Third, the cross-sectional nature of the study made it difficult to establish causal relationship between risk perception and mental health disorders. Last, the subjects' self-reports in the current study were subject to recall bias.

Regardless of the mentioned limitations, our data do provide information regarding psychological impact of acute public events on dialysis patients. Our results urge the necessity of psychotherapeutic interventions for some patients during the current public health event.

## Data Availability Statement

The original contributions presented in the study are included in the article/supplementary material, further inquiries can be directed to the corresponding authors.

## Ethics Statement

The studies involving human participants were reviewed and approved by Review Board of Ruijin Hospital. The patients/participants provided their written informed consent to participate in this study.

## Author Contributions

Z-HY wrote the manuscript. X-TP, YC, LW, Q-XC, YZ, and Y-JZ collected the data. Y-XC and Z-HY designed the study and analyzed the data. X-NC and Y-XC reviewed the manuscript and approved the submission. All authors contributed to the article and approved the submitted version.

## Conflict of Interest

The authors declare that the research was conducted in the absence of any commercial or financial relationships that could be construed as a potential conflict of interest.
